# Synergistic effects through targeting the PI3K and IGFR pathways in treating lung cancer carrying activation alterations along the PI3K pathway

**DOI:** 10.1016/j.tranon.2026.102753

**Published:** 2026-04-03

**Authors:** Mohamed Abd El-Salam, Wu Chen, Yan Tang, Ting Rao, Xuejia Kang, Lifang Sun, Tiegang Han, Pengyu Chen, Matthew Mossanen, Fan Cheng, Chun Yang, Chong-Xian Pan

**Affiliations:** a Department of Medicine, Brigham and Women’s Hospital, Boston, MA, USA; b VA Boston Healthcare System, Boston, MA, USA; c Institute for Research in Biomedicine (IRB) Barcelona, The Barcelona Institute of Science and Technology, Barcelona, Spain; d Department of Pharmacognosy, Faculty of Pharmacy, Delta University for Science and Technology, Gamasa, Egypt; e GIHDiNuC Research Group, Department of Health, School of Health Sciences of TecnoCampus, Pompeu Fabra University, Barcelona, Spain; f Renmin Hospital of Wuhan University, Wuhan, China; g Material Engineering, Auburn University, Auburn, AL, USA; h Department of Urology, Mass General Brigham, Harvard Medical School, Boston, MA, USA; i Department of Psychiatry, Harvard Medical School, Boston, MA, USA

**Keywords:** Lung cancer, PI3K/AKT pathway, PIK3CA mutation, IR/IGF-1R signaling, Ceritinib, Drug resistance, Ketogenic diet

## Abstract

**Purpose:**

Alterations in the PI3K/AKT pathway occur in over 60 % of lung squamous cell carcinoma and approximately 20 % of lung adenocarcinoma, driving tumor progression and therapeutic resistance. While PI3K-targeted therapies suppress proliferation in PIK3CA-mutant non-small cell lung cancer (NSCLC), their clinical impact is limited due to compensatory activation of insulin receptor (IR) and insulin-like growth factor-1 receptor (IGF-1R) signaling. This study aimed to determine whether dual blockade of PI3K and IR/IGF-1R signaling could overcome this adaptive resistance.

**Methods:**

We assessed the effects of combining ceritinib, an ALK and IR/IGF-1R inhibitor, with a PI3K/AKT inhibitor in PIK3CA E545K-mutant H460 NSCLC cells. Functional assays included colonyformation, woundhealing, Matrigel invasion, and Western blot analyses . Therapeutic efficacy was further evaluated in a patient-derived xenograft (PDX) model of lung squamous cell carcinoma harboring the PIK3CA E545K mutation. Transcriptomic profiling using RNA-seq was conducted to identify resistance-associated pathways, and key findings were validated by qRT-PCR.

**Results:**

Genetic ablation of PIK3CA markedly reduced proliferation. Dual inhibition of PI3K and IR/IGF-1R signaling resulted in strong synergistic anti-proliferative, anti-migratory, and anti-invasive effects in vitro. In the PDX model, the combination therapy significantly prolonged overall survival without additional systemic toxicity. Transcriptomic analysis revealed activation of the TNF-α/NF-κB axis as a potential mechanism of acquired resistance.

**Conclusion:**

Concurrent targeting of PI3K and IR/IGF-1R signaling effectively overcomes adaptive resistance in PIK3CA-mutant NSCLC, supporting the rationale for further clinical evaluation of this combined therapeutic strategy.

## Highlights

• Dual PI3K + IR/IGF-1R inhibition showed strong synergy, suppressing proliferation, migration, and invasion in PIK3CA-mutant NSCLC cells.
• Combination treatment significantly extended survival in a PIK3CA E545K-mutant lung squamous PDX model.
• No additional systemic toxicity was observed with the combination therapy in vivo.
• TNF-α/NF-κB signaling emerged as a key resistance mechanism, identified through transcriptomic profiling.

## Graphical abstract

**Figure:**
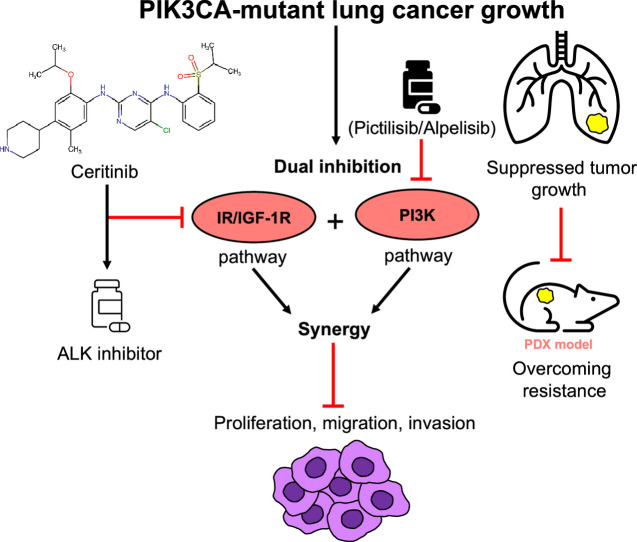

## Funding

This project was supported by the NCI U54 grant (Grant No: U54 CA233306; Pan as a co-PI); VA Merit (Grant No: 1 I01 CX002247-01 and I01 BX006166; PI: Pan)

## Introduction

Non-small cell lung cancer (NSCLC) accounts for approximately 80-85 % of all lung cancer cases and remains the leading cause of cancer-related mortality in the United States and worldwide despite recent advances in targeted therapies and immunotherapy [[1], [2], [3]]. To date, many targeted therapeutic agents have been approved for the treatment of NSCLC. These drugs target the epidermal growth factor receptor (*EGFR*), anaplastic lymphoma kinase (*ALK*), c-ROS oncogene 1 (*ROS1*), the Mesenchymal Epithelial Transition factor (*Met*), rearranged during transfection gene (*RET*), BRAF, neurotrophic receptor tyrosine kinase (*NTRK*), neuregulin 1 (*NRG1*), Kirsten rat sarcoma viral oncogene homolog (*KRAS*), and Human epidermal growth factor receptor 2 (*HER2*) with several more under development. Most of these gene alterations occur in a small subset of NSCLC cases, mainly in lung adenocarcinoma (LADC), and rarely in lung squamous cell carcinoma (LSCC). Of those targeted therapeutic agents, the ALK inhibitor ceritinib is also a strong inhibitor of the insulin receptor (*IR*) and the insulin-like growth factor 1 receptor (*IGF-1R*)[4] achievable at steady-state drug concentrations of approximately 2 μM in clinical settings [5]. The Phosphoinositide 3-kinase (PI3K)/AKT pathway is the most common pathway that is activated in many cancers, especially in squamous NSCLC. However, targeted therapies against the PI3K/AKT pathway are only approved in a few malignancies [6,7] and demonstrates limited efficacy in NSCLC [[8], [9], [10]].

During the treatment of a PI3K/AKT inhibitor, hyperglycemia is a common side effect. For instance, 64 % of patients develop hyperglycemia, and over 36 % experience grade 3 or higher hyperglycemia when treated with alpelisib [11], an oral inhibitor of the PI3K alpha subunit (p110α) approved by the US Food and Drug Administration. Hyperglycemia can stimulate insulin secretion, activating the insulin receptor (IR) pathway and potentially mitigating the anti-tumor effect of PI3K/AKT pathway inhibitors. Inhibition of the IR or the use of a ketogenic diet has been shown to significantly potentiate the anti-tumor effect of PI3K/AKT inhibition [12]. We previously demonstrated that the insulin-like growth factor 1 receptor (IGF-1R) pathway was upregulated when bladder cancer patient-derived xenografts (PDXs) were resistant to PI3K/AKT inhibition. Both IR and IGF-1R are frequently upregulated in NSCLC [[13], [14], [15]], with IR and IGF-1R are expressed in approximately 40 % and 80 % of NSCLC cases, respectively [15,16]. Both receptors can activate the PI3K/AKT pathway. Based on these observations, we hypothesized that simultaneous inhibition of the PI3K/AKT pathway and the IR/IGF-1R pathway could produce synergistic anti-tumor effects in NSCLC with activation alterations in the PI3K/AKT pathway. This study aimed to evaluate the efficacy of combining a PI3K/AKT pathway inhibitor with the ALK/IR/IGF-1R inhibitor ceritinib in NSCLC harboring PI3K/AKT pathway activation alterations.

## Materials and methods

### Animals

Immunodeficient female mice (strain: NOD.Cg-Prkdc^SCID^ Il2rg^tm1Wjl^/SzJ, or NSG; RRID: BCBC_4142) were purchased from the Jackson Laboratory (Bar Harbor, ME., USA). All animal experiments were conducted in accordance with the National Institute of Health Guide for the Care and Use of Laboratory and complied with U.S. Veterans Administration and Harvard University guidelines. Every effort was made to minimize both the number of animals used and their discomfort.

### Ethical statement

All experimental procedures were approved by the Institutional Animal Care and Use Committee and Institutional Biosafety Committee of the Veterans Administration Boston Healthcare System (IACUC No. 1577782) prior to initiation of in vivo studies.

### Study materials

Pictilisib (G-9252), ceritinib (C-2086), and alpelisib (A-4477) were purchased from LC Laboratories (Woburn, MA, USA). The lung cancer cell line was obtained directly from the American Type Culture Collection (ATCC) and cultured in the recommended medium for fewer than six months prior to use in experiments; therefore, additional authentication was not performed. The following antibodies were used: rabbit anti-pAkt (Cell Signaling Technology Cat# 9271; RRID: AB_329825), rabbit anti-Akt (Cell Signaling Technology Cat# 9272; RRID: AB_329827), mouse anti-GAPDH (Santa Cruz Biotechnology Cat# sc-32233; RRID: AB_627679), mouse anti-mTOR (Abcam Cat# ab32028; RRID: AB_881283), mouse anti-caspase-3 (Abcam Cat# ab32351; RRID: AB_725946), mouse anti-IGF-1R (Santa Cruz Biotechnology Cat# sc-462; RRID: AB_627781), HRP-conjugated secondary antibodies (Bio-Rad, Cat #: 12010020).

### *In vitro* experiments with lung cancer cells

For the cell viability assay, cells were cultured and treated with varying concentrations (0, 5, 10, 25, and 50 μM) of ceritinib, pictilisib, or their combination for 24 hours and analyzed using the colorimetric WST-1 cytotoxicity assay (Cat# ab65475, Abcam). The combination index of the drugs was calculated using the Chou–Talalay method as described previously [17]. For colony-forming assay, cells were cultured for 15 days before fixed with methanol, stained with 0.005 % crystal violet (#C0775, Sigma Aldrich) and analyzed by inverted microscope (Olympus Corporation, Tokyo, Japan) for the average number of colonies and the number of cells per colony. For the colony-forming assay, cells were cultured for 15 days, fixed with methanol, stained with 0.005 % crystal violet (C0775, Sigma-Aldrich), and analyzed under an inverted microscope (Olympus Corporation, Tokyo, Japan) for the average number of colonies and cells per colony. For the cell migration assay, a straight scratch was made with a sterile pipette tip on the confluent cancer cell monolayer, and cells were then cultured with vehicle control or drug treatments for 24, 48, and 72 hours. Cell migration was assessed by imaging the wound area at each time point with an inverted phase-contrast microscope and quantifying closure using ImageJ software, expressed as the percentage of closure relative to the initial wound width at 0 h. A Transwell invasion assay was performed according to published protocols to evaluate the inhibitory effects of ceritinib, pictilisib, and their combination on cell invasion [[18], [19], [20]].

### Western blot analysis

H460 lung cancer cells were treated with vehicle control, test drugs (0.5 and 1 μM), or their combination for 24, 48, and 72 hours before harvesting. Proteins were separated on 7.5 % Mini-PROTEAN® TGX™ Precast Protein Gels (#4561023, Bio-Rad) at 120 V for 120 minutes. After electrophoresis, proteins were transferred to nitrocellulose membranes and incubated overnight at 4°C with primary antibodies (1:1000), followed by HRP-conjugated secondary antibodies (1:5000) in EveryBlot Blocking Buffer (#12010020, Bio-Rad). Immunoreactive bands were detected using SignalFire™ ECL Reagent (#6883, Cell Signaling Technology) and SuperSignal™ West Femto Maximum Sensitivity Substrate (#34094, Thermo Scientific) and visualized by autoradiography. Band intensity was quantified using ImageJ (RRID: SCR_003070).

### *In vivo* xenograft treatment studies

The patient-derived xenograft (PDX) of lung cancer was provided by the Jackson Laboratory (Model ID: TM00244/LG1235). The presence of a PIK3CA mutation was confirmed by exon sequencing. PDX specimens/cells were implanted subcutaneously into the right flanks of 8-week-old NSG female mice. Tumor volume was calculated as (length × width²) × 0.5. Only mice with successfully implanted tumors reaching ∼200 mm³ were included. Mice were randomized into four treatment groups (N = 8 per group): control, alpelisib alone (25 mg/kg), ceritinib alone (25 mg/kg), or a combination of both drugs (25 mg/kg each). All drugs were administered by oral gavage once daily for two weeks. Tumor volumes and body weights were recorded every two days in a blinded manner. Attrition was monitored throughout the study, and any mouse showing health issues or significant weight loss beyond ethical limits was euthanized. After 3 and 14 days of treatment, three mice from each group were randomly selected for euthanasia. Tumors were collected and fresh-frozen at –80°C for further RNA-seq and qPCR analyses.

### RNA-seq analysis and data processing

RNA sequencing service was provided by Novogene (Sacramento, CA, USA). Tumor tissues were collected, flash-frozen, and total RNA was extracted. Poly-T oligo-attached magnetic beads were used to enrich mRNA. Strand-specific cDNA libraries were prepared through fragmentation, first- and second-strand synthesis (with dUTP or dTTP incorporation depending on library type), end repair, A-tailing, adapter ligation, size selection, PCR amplification, and purification. Library quality was confirmed by Qubit, real-time PCR, and bioanalyzer assays. Pooled libraries were sequenced on an Illumina platform according to effective library concentration and expected data yield.

### Bioinformatics pipeline

Raw FASTQ files underwent quality control (adapter removal, filtering of low-quality and poly-N reads), and quality metrics (Q20, Q30, GC content) were assessed. Reads were aligned to the reference genome using Hisat2 v2.0.5, with splice junction database support. Gene expression was quantified with featureCounts, normalized as FPKM to account for gene length and sequencing depth. DESeq2 was used for differential expression analysis in biological replicates, and edgeR was applied in cases without replicates. Multiple testing correction was performed with the Benjamini–Hochberg method, with significance defined as adjusted *p* ≤ 0.05.

### Pathway and enrichment analysis

Functional enrichment was carried out with ClusterProfiler, testing enrichment across GO (RRID:SCR_002811), KEGG (RRID:SCR_012773), Reactome (RRID:SCR_003485), Disease Ontology (RRID:SCR_000476), and DisGeNET (RRID:SCR_006178) databases (corrected p ≤ 0.05). In addition, Gene Set Enrichment Analysis (GSEA) was performed to assess the enrichment of predefined gene sets across treatment and resistance conditions.

### Additional analyses

Variants were called using GATK v4.1.1.0 and filtered according to best-practice parameters. Alternative splicing events were detected with rMATS v4.1.0. Protein–protein interaction networks were constructed via the STRING database. Fusion transcripts were identified with STAR-Fusion v1.9.0, based on STAR alignments with downstream filtering.

### Real-Time qRT-PCR analysis of TNFAIP3/NF-κB expression levels

In each tumor tissue sample, four distinct regions were randomly collected and pooled. Total RNA was then extracted using the QIAwave RNA Mini Kit (250) (#74536, QIAGEN) according to the manufacturer’s instructions. After reverse transcription, complementary DNA (cDNA) was amplified using the specific primers listed below and Power SYBR Green PCR Master Mix (#4367659, Applied Biosystems). The β-actin mRNA level served as an internal normalization control. Primer sequences were as follows: β-actin: CACCATTGGCAATGAGCGGTTC (forward) and AGGTCTTTGCGGATGTCCACGT (reverse); TNFAIP3 human: CTCAACTGGTGTCGAGAAGTCC (forward) and TTCCTTGAGCGTGCTGAACAGC (reverse). Relative mRNA expression was quantified using the 2−ΔΔCT method and normalized to β-actin levels.

#### Statistical analysis

Experiments were performed in independent technical triplicates, and data are expressed as the mean ± standard deviation (S.D.). Analyses were conducted using GraphPad Prism Software (version 9.0.2). Comparisons between two groups were performed using the Student’s t-test, while multiple-group comparisons employed one-way analysis of variance (ANOVA) followed by Tukey’s post-hoc test. CalcuSyn Version 2.22 was used to calculate the combination index.

## Results

### Alteration along the PI3K/AKT pathway in NSCLC

**Fig. 1:**
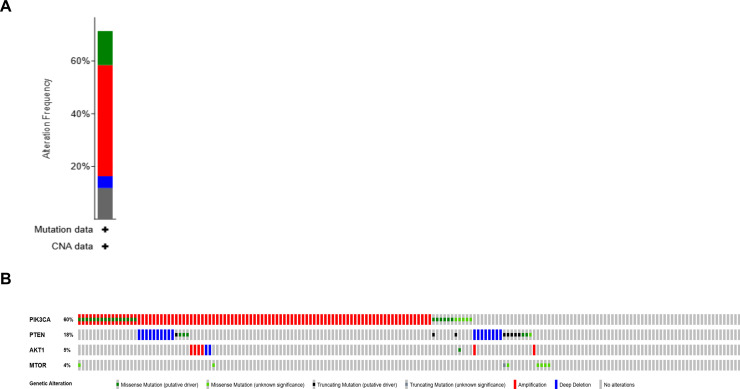

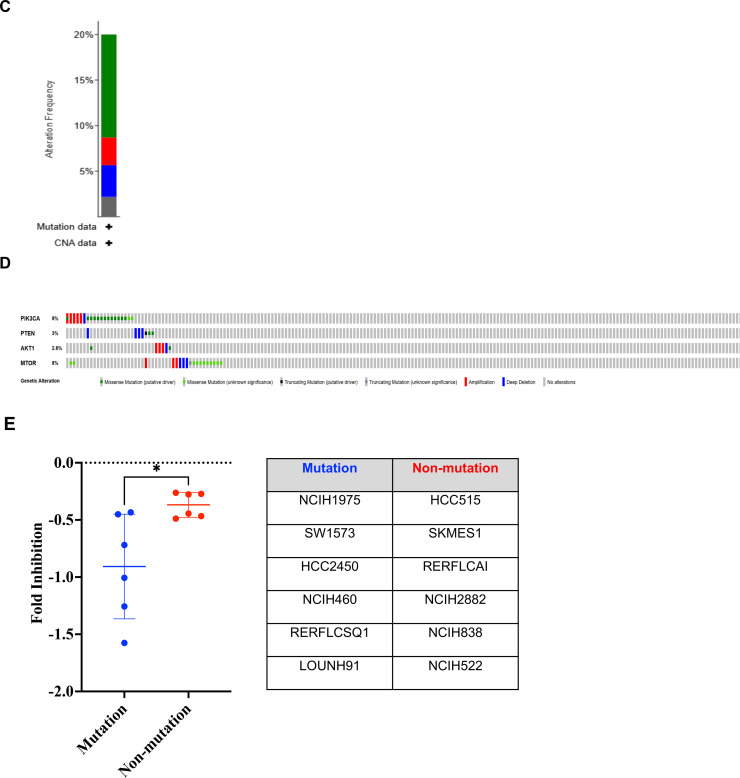
Genomic alteration landscape in the PI3K/AKT pathway from The Cancer Genome Atlas (TCGA) dataset, analyzed via cBioPortal (cbioportal.org) in lung cancer. Alterations are observed in 71 % of lung squamous cell carcinomas (LSCCs; panels A, B) and 20 % of lung adenocarcinomas (LADCs; panels C, D), involving PIK3CA, PTEN, AKT1, and/or mTOR. Panels B and D show the nature of these alterations: PIK3CA, AKT1, and mTOR are predominantly affected by activating mutations or gene amplifications, whereas PTEN alterations are mostly deletions, truncations, or loss-of-function mutations. (E) Impact of PIK3CA alterations on lung cancer cell growth. DepMap CRISPR knockout data show that PIK3CA inactivation significantly reduces growth in cell lines harboring activating PIK3CA mutations compared to six lines without such mutations (log2 inhibition −0.907 vs −0.368, p = 0.0186; data from DepMap.org). The analyzed cell lines are shown in the right panel.

We analyzed The Cancer Genome Atlas (TCGA) database through cBioPortal to determine the prevalence of alterations along the PI3K/AKT pathway in lung cancer. Alterations in at least one of the 17 genes of this pathway were identified in 88.8 % of lung squamous cell carcinoma (LSCC; Supplemental Information SI-1A) and 42.8 % of lung adenocarcinoma (LADC; SI-1C). Among these, 71 % of LSCCs (Fig. 1A, B) and 20 % of LADCs (Fig. 1C, D) harbored at least one mutation in PIK3CA, PTEN, AKT1, and/or mTOR-gene for which FDA-approved targeted drugs are available. Although some alterations (deep deletions of AKT1 and mTOR) are unlikely to be functional drivers, most others are considered putative drivers. For instance, all 256 LSCC cases (100 %) carrying PIK3CA alterations (SI-1B) were classified as putative drivers, comprising 59 activating mutations, 176 gene amplifications, and 21 cases with combined activating mutations and amplifications. Among 45 LADC cases with PIK3CA alterations (SI-1D), 96 % (43 cases) were classified as putative drivers, including 29 activating mutations, 11 gene amplifications, and 3 cases with both. We previously showed that PIK3CA activating mutations and amplifications act as molecular drivers and represent viable therapeutic targets [21]. For the tumor suppressor gene PTEN, predominant alterations include deletions and truncations (SI-1B, 1D). Although the frequency is lower in LADC (20 %) than LSCC (71 %), these events remain more prevalent than many other targetable alterations such as ALK, ROS1, MET, RET, and NTRK.

### Alterations along the PI3K/AKT pathway as drivers of NSCLC

Next, we assessed whether these alterations act as functional drivers in lung cancer. Using the DepMap database, we analyzed the effect of PI3K/AKT pathway inhibition on cell proliferation. CRISPR-mediated knockout of PIK3CA resulted in significantly greater growth inhibition in cell lines carrying PIK3CA activating mutations compared with six cell lines lacking such mutations (log₂ inhibition: −0.907 vs. −0.368, p = 0.0186; depmap.org, Fig. 1E).

### Synergistic effect between ceritinib and a PI3K/AKT inhibitor in suppressing cell growth and colony formation

An NSCLC cell line (H460) harboring the E545K activating mutation of PIK3CA was selected to evaluate drug–drug interactions between ceritinib and pictilisib, an oral PIK3CA inhibitor. In the clinic, steady-state drug concentrations of ceritinib and pictilisib are approximately 2 μM [5,22]. To enhance clinical translatability, we used concentrations readily achievable in patients.

**Fig. 2:**
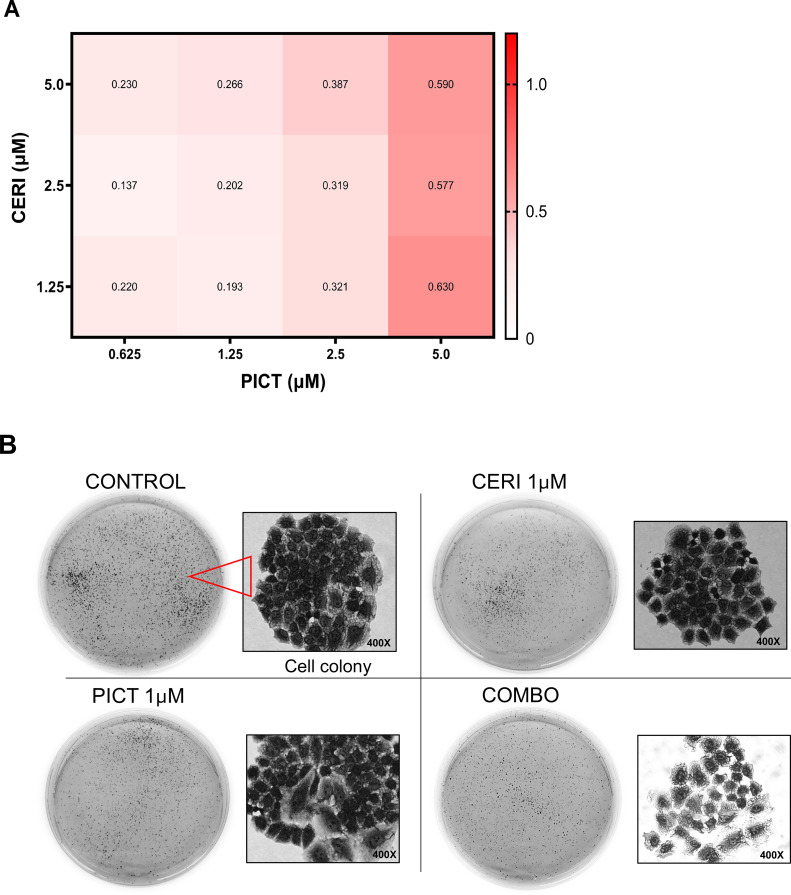

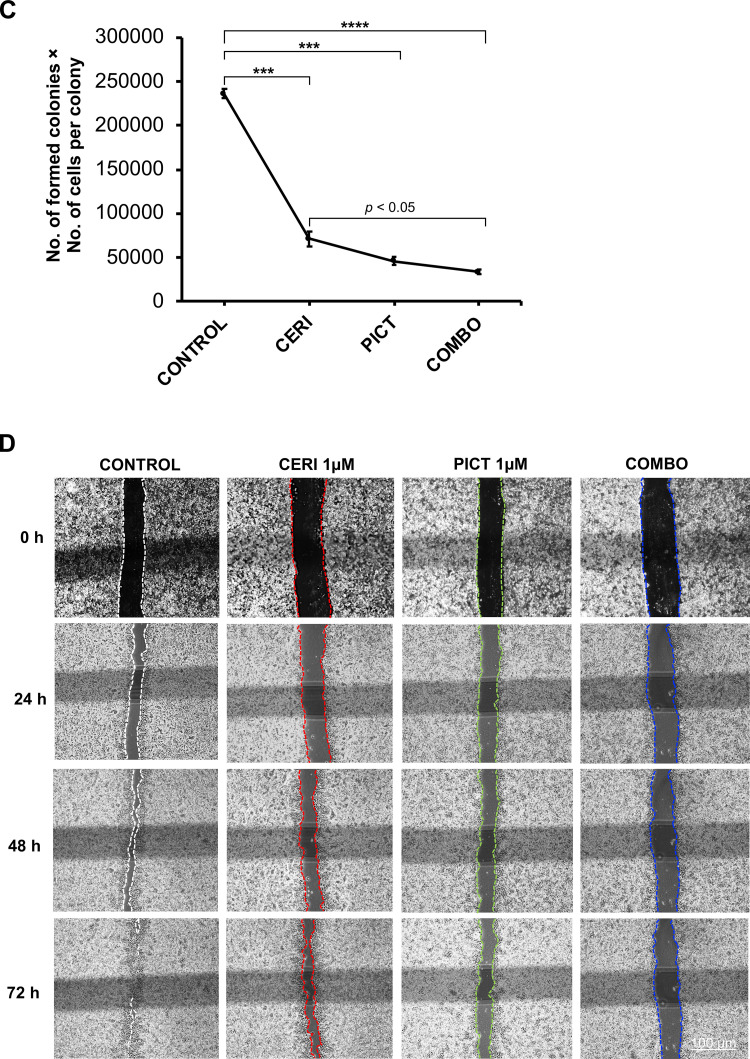

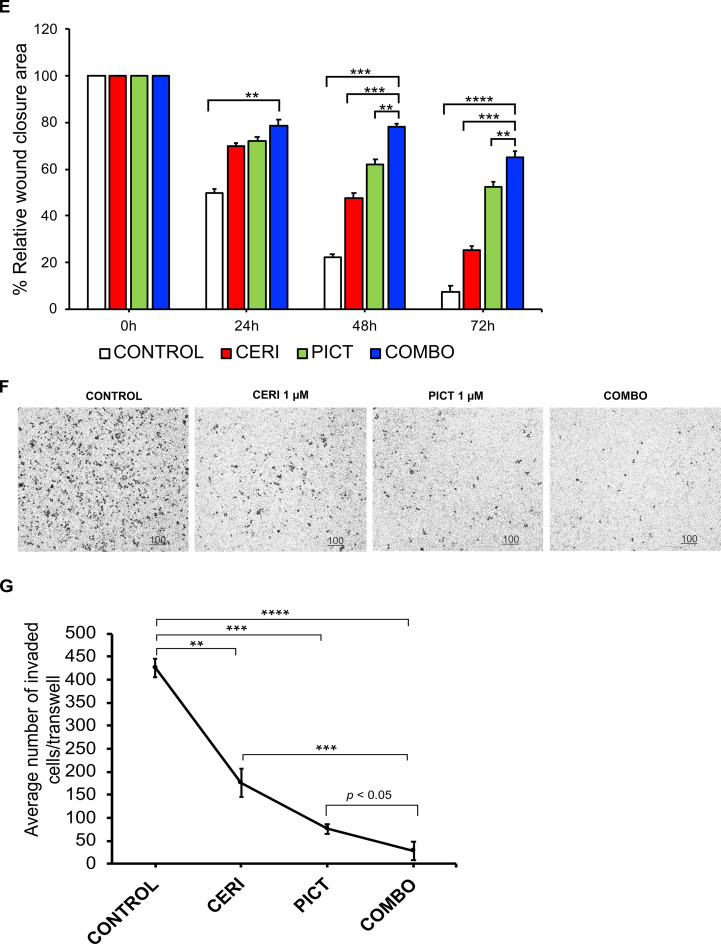
Ceritinib and pictilisib inhibit lung cancer cell growth, colony formation, migration, and invasion in vitro. (A) Drug-drug synergistic interaction. Combination index (CI) values indicate additivity (0.9–1.1), antagonism (>1.1), synergy (<0.9), and strong synergy (<0.7). Ceritinib and pictilisib showed strong synergy at most concentrations, with apparent antagonism only at very high doses likely due to G₀-phase cell survival. (B, C) Colony formation. Clonogenic assays in H460 cells show reduced colony number and size with either drug and the greatest reduction with the combination (*p* < 0.001). Total cell number was calculated by multiplying average colonies per dish by cells per colony. (D, E) Cell migration. Scratch assays were imaged at 0, 24, 48, and 72 h. Relative wound closure was quantified by ImageJ. At 72 h, closure was 34.9 % with the combination, 47.6 % with pictilisib, 74.7 % with ceritinib, and 92.8 % in controls (combination vs ceritinib *p* < 0.001, pictilisib *p* < 0.01, control *p* < 0.0001). (F, G) Cell invasion. Transwell assays imaged at 24 h show fewer invading cells after drug treatment. Relative to control, ceritinib, pictilisib, and combination reduced invasion by 58.6 % (*p* < 0.01), 82.1 % (*p* < 0.001), and 93.2 % (*p* < 0.0001), respectively. Combination produced significantly fewer invading cells than ceritinib (*p* < 0.001) or pictilisib (*p* < 0.05). **p < 0.01; ***p < 0.001; ****p < 0.0001.

First, we determined the combination index (CI), where CI < 1 indicates synergy and CI < 0.7 denotes strong synergy [23]. Strong synergy was consistently observed across all drug combinations (Fig. 2A). We then conducted a colony-forming assay. Cells treated with either drug alone or the combination produced fewer colonies and fewer cells per colony than untreated controls (Fig. 2B, C). The reduction was statistically significant for the combination versus ceritinib alone (*p* < 0.05, Fig. 2C), while no additional decrease was observed relative to pictilisib alone.

### Synergistic effect between ceritinib and pictilisib in inhibiting cell migration and invasion

We next investigated whether the treatments affect cell migration and invasion, key processes in metastasis. Although single-agent treatments inhibited migration, the combination was most effective (Fig. 2D, 2E). At 72 hours, migration was reduced to 34.89 % of the covered area in the combination group, compared with 47.63 % for pictilisib, 74.68 % for ceritinib, and 92.83 % for the control. Inhibition by the combination was significantly greater than ceritinib (*p* < 0.001), pictilisib (*p* < 0.01), and control (*p* < 0.0001). Trans-well assays showed invasion decreases of 58.6 % (*p* < 0.01), 82.1 % (*p* < 0.001), and 93.2 % (*p* < 0.001) for ceritinib, pictilisib, and the combination, respectively. Invasion in the combination group was significantly lower than in ceritinib (*p* < 0.001) and pictilisib (*p* < 0.05) groups (Fig. 2F, G). These findings indicate that the combination therapy provides a stronger inhibitory effect than ceritinib alone and enhances anti-migratory and anti-invasive activity, supporting a synergistic interaction between PI3K and IR/IGF-1R inhibition.

### Inhibition of the PI3K/AKT pathway by ceritinib, pictilisib and combination treatments

**Fig. 3:**
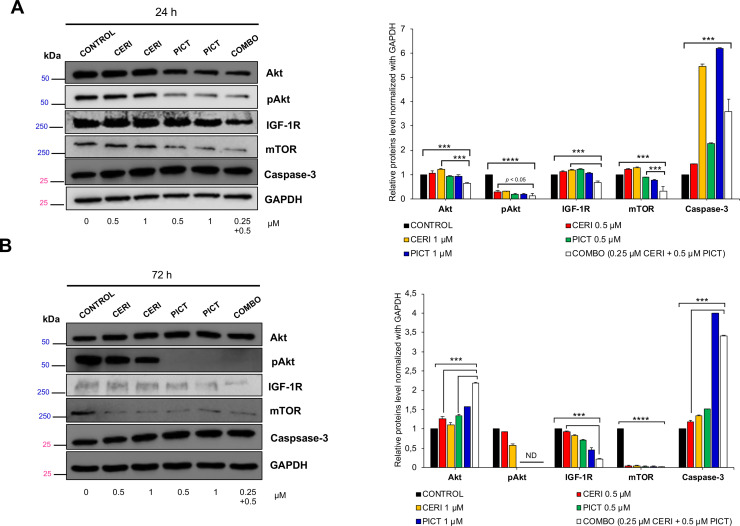
Western blot analysis of IGF-1R, PI3K/AKT pathway proteins and Caspase-3 in H460 cells. Cells were treated with ceritinib, pictilisib, or their combination for 24 h (A) or 72 h (B) at the indicated concentrations. Membranes were probed with the relevant primary antibodies and HRP-conjugated secondary antibodies (anti-rabbit or anti-mouse). GAPDH served as the loading control. Band intensities were quantified with ImageJ; ND indicates not detected. Combination treatment suppressed PI3K/AKT/mTOR signaling and reduced IGF-1R expression. Expression of caspase-3, an apoptosis marker, was relatively activated in the combination-treatment group compared with the control or singledrug groups. ***p < 0.001; ****p < 0.0001.

IGF-1R and IR regulate downstream signaling via the PI3K/AKT/mTOR pathway. To determine how treatments modulate signaling, we performed Western blots on H460 cells using sub-IC₅₀ concentrations (doses below the half-maximal inhibitory concentration) to evaluate the direct effects of the drugs on signaling proteins while minimizing non-specific toxicity and stress responses. Specimens were collected at 24 and 72 hours to evaluate immediate and sustained effects. At 24 hours (Fig. 3A), pAkt levels decreased in all treatment groups, indicating pathway suppression. IGF-1R was also reduced in the combination group but not with single agents. Interestingly, a modest rise in total mTOR and IGF-1R occurred with ceritinib alone, likely reflecting transient feedback to partial ALK inhibition. By 72 hours (Fig. 3B), this compensatory effect subsided: pAkt remained suppressed in pictilisib and combination groups, mTOR was downregulated across all groups, and IGF-1R suppression was most pronounced in the combination group. Caspase-3, an apoptosis marker, was relatively activated in the combination group than in untreated or single-drug groups. The enhanced suppression of IGF-1R and activation of caspase-3 in the combination group further supports the cooperative mechanism underlying the observed synergy.

### *In vivo* effects on tumor growth with a patient-derived xenograft

**Fig. 4:**
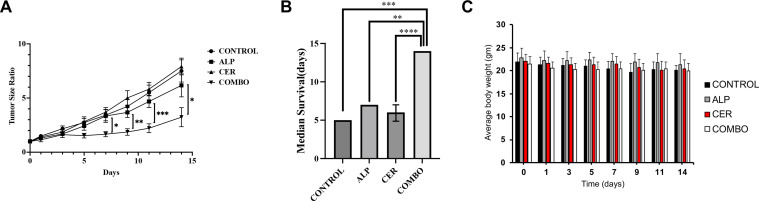
Inhibition of tumor growth in LSCC patient-derived xenografts (PDX). PDX LG1235, harboring the activating PIK3CA E545K mutation in exon 9, was used. When tumors reached ∼200 mm³, NSG mice were randomized into four treatment groups (n = 8 per group): vehicle control, alpelisib (25 mg/kg), ceritinib (25 mg/kg), or the combination (25 mg/kg each). Drugs were administered once daily via oral gavage for two weeks. Tumor volumes and body weights were recorded every two days in a blinded manner. Mice were euthanized when tumors reached ∼5 × baseline volume. (A) Tumor growth. Single-agent alpelisib or ceritinib did not significantly suppress tumor growth, whereas combination treatment (COMBO) significantly slowed growth compared to control (CONTROL), alpelisib (ALP), or ceritinib (CER) groups. *p < 0.05, **p < 0.01, ***p < 0.001 (ANOVA). (B) Median survival. Time to reach 3 × baseline tumor volume is shown. By day 14, all control, ALP, and CER mice (except one in ALP) reached this benchmark, whereas only 2/8 mice in the combination group did. Tumor doubling times were 5.0 ± 0.0, 7.0 ± 0.0, and 6.0 ± 1.0 days for CONTROL, ALP, and CER, respectively; the COMBO group did not reach 3 × baseline, and 14 days was used as the endpoint for euthanasia. **p < 0.01; ***p < 0.001; ****p < 0.0001. (C) Body weight changes. No significant differences in body weight were observed among groups during treatment. ALP, alpelisib; CER, ceritinib; COMBO, alpelisib + ceritinib.

Next, we determined whether the *in vitro* findings can be translated into *in vivo* effects with a patient-derived LSCC xenograft (PDX). We previously reported that these PDXs recapitulate clinical presentation and genomic alterations of patient cancers [24,25]. We used the LG1235 PDX which is a LSCC PDX developed from a 62-year-old Caucasian female smoker and contains the PIK3CA E545K activation mutation in exon 9. We previously showed that different PIK3CA inhibitors had comparable anti-tumor activities in PDXs [21,25,26]. Given the market withdrawal of several inhibitors, we used alpelisib, an FDA-approved oral PIK3CA inhibitor for the treatment of metastatic breast cancer harboring an activation alteration of PIK3CA, AKT or PTEN. When PDXs reached ∼ 200 mm^3^, mice were randomized into vehicle control, alpelisib alone, ceritinib alone, or the combination (eight mice per group). Drugs were administered orally once daily. Neither alpelisib nor ceritinib alone significantly inhibited tumor growth, whereas the combination produced marked suppression (Fig. 4A). Mice were euthanized when tumors reached fivefold baseline (∼1,000 mm³) or developed ulceration; the experiment ended at Day 14. Because ulceration compromised measurements beyond a threefold increase, we calculated survival as time to triple baseline volume. At Day 14, nearly all mice in control, alpelisib, and ceritinib groups exceeded triple baseline, whereas only two of eight mice in the combination group did (SI-2). The time for tumor volume to increase three-fold from baseline was 5.0 ± 0.0 days for the control group, 7.0 ± 0.0 days for the alpelisib-treated group, and 6.0 ± 1.0 days for the ceritinib-treated group. In the combination-treatment group, the three-fold increase was not reached by the majority of animals during the observation period (only 2 mice eventually reached it), and the difference was significant versus the other three groups (*p* < 0.01) (Fig. 4B). No significant changes in body weight (Fig. 4C), behavior, or food/water intake were observed, indicating low systemic toxicity.

### Upregulation of TNF-alpha pathway is associated with resistance to treatments

**Fig. 5:**
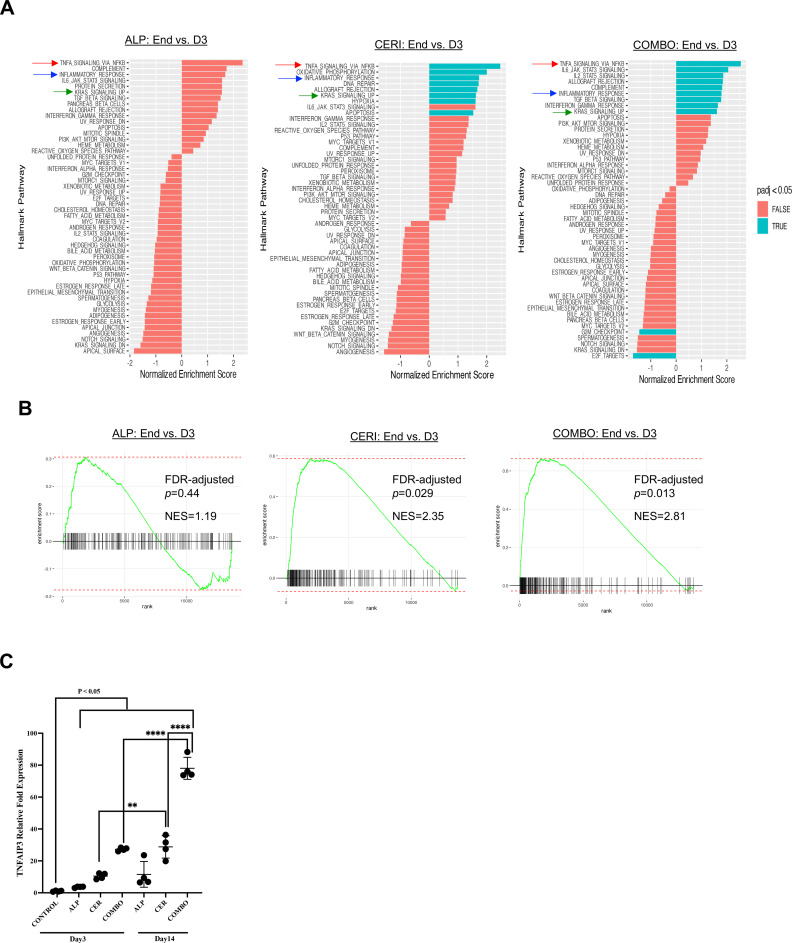
Pathway alterations associated with secondary resistance. (A) RNA-seq and GSEA analyses of PDX specimens collected after three days of treatment and at the time of secondary resistance reveal upregulation of the TNF-α signaling pathway via NF-κB, which ranks highest when PDXs became resistant to all three treatments. Inflammatory response and KRAS signaling pathways were also upregulated upon resistance. (B) TNF-α/NF-κB pathway upregulation. Although this pathway was not significantly altered in alpelisib-resistant PDXs (q = 0.44, NES = 1.19), it was significantly upregulated in ceritinib- and combination-resistant PDXs (q/NES = 0.029/2.35 and 0.013/2.81, respectively). (C) TNFAIP3 expression. qRT-PCR confirmed increased TNFAIP3, a key gene in the TNF-α signaling pathway, in resistant tumors. Expression was elevated after 3 days of treatment and further increased at resistance, particularly in ceritinib and combination groups. In contrast, TNFAIP3 changes in alpelisib-treated PDXs were not significant. These results are consistent with the GSEA findings. ALP, alpelisib; CER, ceritinib; COMBO, drug combination; END, time of secondary resistance; D3, day 3.

To elucidate mechanisms of resistance, mice were euthanized on Day 3 (sensitive phase) and at endpoint (resistant phase). PDXs were harvested for RNA sequencing, and gene-set enrichment analyses (GSEA) were performed (SI-3). Pathways consistently upregulated included TNF-α, inflammatory response, and KRAS signaling (Fig. 5 A). TNF-α upregulation ranked highest across all three resistant PDXs, though it was not statistically significant in alpelisib-resistant tumors (NES/adjusted p: 1.17/0.44, alpelisib; 2.35/0.028, ceritinib; 2.81/0.013, combination, Fig. 5B). We then performed qRT-PCR analysis to validate and confirm the RNAseq and GSEA findings (Fig. 5C). TNFAIP3 expression was slightly elevated at Day 3 but markedly increased in ceritinib- and combination-resistant PDXs, whereas alpelisib groups showed no significant change, which were consistent with the RNAseq and GSEA findings.

#### Synergy between ceritinib and PI3K/AKT pathway inhibitors across multiple cancer types

Finally, we queried publicly available databases to determine whether ceritinib–PI3K/AKT inhibitor synergy extends beyond NSCLC. Evidence of synergy was observed in all five tested cell lines of diverse tissue origin across four different PI3K/AKT inhibitors (Table 1). No antagonistic interactions were identified, suggesting that the synergistic effect is likely conserved across multiple cancer types.

**Table 1:** Synergy between ceritinib and PI3K/AKT/mTOR pathway inhibitors.

Drug name	Drug target	Cell line	Cell line tissue origin	Bliss*	Drug-drug interaction	source
MK-2206	AKT	SMS-CTR	Embryonal rhabdomyosarcoma	8.45	synergy	https://drugcomb.fimm.fi/
Omipalisib	PI3K/AKT	SMS-CTR	Embryonal rhabdomyosarcoma	10.16	synergy	https://drugcomb.fimm.fi/
Everolimus	mTOR	SMS-CTR	Embryonal rhabdomyosarcoma	16.55	synergy	https://drugcomb.fimm.fi/
MK-2206	AKT	SU-DIPG-XIII	Diffuse intrinsic pontine glioma	6.26	synergy	https://drugcomb.fimm.fi/
Omipalisib	PI3K/AKT	SU-DIPG-XIII	Diffuse intrinsic pontine glioma	17.58	synergy	https://drugcomb.fimm.fi/
Everolimus	mTOR	SU-DIPG-XIII	Diffuse intrinsic pontine glioma	13.54	synergy	https://drugcomb.fimm.fi/
MK-2206	AKT	RD	Rhabdomyosarcoma	12.68	synergy	https://drugcomb.fimm.fi/
Omipalisib	PI3K/AKT	RD	Rhabdomyosarcoma	18.63	synergy	https://drugcomb.fimm.fi/
Everolimus	mTOR	RD	Rhabdomyosarcoma	15.83	synergy	https://drugcomb.fimm.fi/
MK-2206	AKT	RH36	Embryonal rhabdomyosarcoma	9.862	synergy	http://drugcombdb.denglab.org
GSK-2126458	PI3K/AKT	RH36	Embryonal rhabdomyosarcoma	11.855	synergy	http://drugcombdb.denglab.org
Everolimus	mTOR	RH36	Embryonal rhabdomyosarcoma	19.425	synergy	http://drugcombdb.denglab.org
GSK-2126458	PI3K/AKT	CTR	Rhabdomyosarcoma	20.505	synergy	http://drugcombdb.denglab.org
Everolimus	mTOR	CTR	Rhabdomyosarcoma	15.796	synergy	http://drugcombdb.denglab.org
MK-2206	AKT	RD	Rhabdomyosarcoma	14.789	synergy	http://drugcombdb.denglab.org
GSK-2126458	PI3K/AKT	RD	Rhabdomyosarcoma	21.734	synergy	http://drugcombdb.denglab.org
Everolimus	mTOR	RD	Rhabdomyosarcoma	18.468	synergy	http://drugcombdb.denglab.org

*Excess over Bliss calculations measure whether the observed combinatorial effects at given concentrations are above or below predicted additivity, where a score of 0 indicates additive, above 0 for synergistic and less than 0 for antagonistic, according to Liu et al., 2018 [1].

1. Liu Q, Yin X, Languino LR, Altieri DC: Evaluation of drug combination effect using a Bliss independence dose-response surface model. *Stat Biopharm Res* 2018, 10:112-122.

## Discussion

Over the past decade, targeted therapy has reshaped the treatment landscape for non-small-cell lung cancer (NSCLC), with more than a dozen agents gaining regulatory approval. Nonetheless, fewer than half of lung adenocarcinoma (LADC) cases and only a small fraction of lung squamous cell carcinoma (LSCC) are eligible for these therapies. Our results provide compelling evidence that the PI3K/AKT pathway is a therapeutically actionable target in NSCLC, particularly in LSCC.

The PI3K/AKT pathway is a central oncogenic driver in many malignancies, including NSCLC. It is a downstream signaling pathway of numerous cell surface receptors that promotes cell proliferation, survival, and metabolism [8]. Moreover, this pathway is a key link that modulates multidrug resistance in diverse types of cancers [27]. Mutations or alterations in genes encoding components of this pathway, including *PIK3CA*, can lead to its constitutive activation and drive tumor growth. Nearly 90 % of LSCCs and 40 % of LADCs contain at least one alteration along this pathway (SI-1), of which approximately 70 % of LSCC (Fig. 1A and B) and 20 % of LADC (Fig. 1C and D) harbor alterations at PIK3CA, PTEN, AKT1 and/or mTOR genes that can currently be targeted by FDA-approved drugs. Most of these alterations cause constitutive activation of this pathway in that they are either activation mutation or amplification of oncogenic genes (PIK3CA, AKT1 and mTOR), or deletion, mutation or truncation of the tumor suppressor gene PTEN.

So far, many studies targeting this PI3K/AKT pathway have been conducted. Except in breast cancer, lymphoma and kidney cancer, medications targeting the pathway alone in other cancers, including NSCLC, have shown limited efficacy or have developed secondary resistance soon after an initial response [10,28]. Resistance is often attributed to the activation of compensatory signaling pathways [29]. Understanding these mechanisms of resistance is crucial to improving treatment efficacy and overcoming limitations associated with targeted therapy.

Our previous studies with PI3K inhibitors in bladder cancer revealed that the IGF-1R pathway might play a role in secondary resistance to inhibitors of targeting the PI3K/AKT pathway [21,30]. Numerous studies have explored the implication of IGF-1R in NSCLC. IGF-1R is frequently upregulated in NSCLC, including over 80 % of LSCC [16,31]. High IGF-1R expression in NSCLC and elevated levels of circulating IGF-1 are associated with poor prognosis [32,33]. IGF-1R modifies the tumor microenvironment that facilitates lung cancer metastasis and progression [34]. To date, two major strategies have been investigated to interfere with IGF-1R pathway signaling in cancers: the use of monoclonal antibodies directed against the IGF-1R extracellular domain and small-molecule inhibitors targeting its intracellular kinase domain [35]. The latter approach can reduce signaling pathways without requiring receptor internalization. IGF-1R inhibitors have shown some clinical efficacy [36], but overall, their efficacy is disappointing [37].

Our study demonstrates the therapeutic potential of combining a PI3K/AKT inhibitor with ceritinib, which inhibits IR, IGF-1R and ALK. Ceritinib has been approved for NSCLC harboring an ALK activation alteration, commonly the translocation and creation of a fusion protein between the echinoderm microtubule-associated protein-like 4 (EML4) gene and the ALK gene [38]. ALK rearrangements occur in approximately 5 % of advanced NSCLC [39], the vast majority of which are adenocarcinoma (>95 %) in never- or former- smoker (>95 %) [40]. In this study, ceritinib is not used as an ALK inhibitor but rather as an IGF-1R and IR inhibitor. Ceritinib is a strong inhibitor of IGF-1R and IR with the IC_50_ of 8 and 7 nM, respectively [4]. This combination is built upon a solid rationale and addresses a critical unmet needs in NSCLC, especially LSCC. As discussed above, the vast majority of NSCLCs eligible for targeted therapy are LADC. Over 90 % of LSCC harbor an alteration along the PI3K/AKT/mTOR pathway (SI-1) and over 80 % of LSCC have overexpression of IGF-1R [41], suggesting that, if successful, most LSCC patients can be treated with this combination. Our studies show that a PI3K/AKT pathway inhibitor and ceritinib showed synergistic effects in inhibiting cell proliferation (Fig. 2 A and B) and colony formation (Fig. 2 C and D), suppressing cell migration (Fig. 2 E and F) and invasion (Fig. 2 G and H), and reducing tumor growth in our PDX model carrying a PIK3CA activation mutation (Fig. 4 A and B). This combination seems to be well tolerated well with no observed systemic toxicity (Fig. 4 C). More importantly, ceritinib appears to synergize with other inhibitors of the PI3K/AKT pathway in multiple cell lines across multiple histological types (Table 1). Although pictilisib alone showed strong activity in some in vitro assays, the combination with ceritinib resulted in enhanced pathway inhibition and superior efficacy in the PDX model, supporting the therapeutic advantage of dual targeting of PI3K and IR/IGF-1R signaling. The in vitro evaluation was primarily conducted in the PIK3CA-mutant NSCLC cell line H460, which was selected for detailed mechanistic studies. We also tested the combination in NCI-H2228; however, the treatment showed limited synergy in this line, likely due to its distinct molecular profile (EML4-ALK fusion). Our future studies will expand this evaluation to additional PIK3CA-mutant NSCLC cell lines to further support the generalizability and translational relevance of this therapeutic strategy.

Another significant finding of our study is the modulation of the TNF-α/NF-κB signaling pathway by dual inhibition of PI3K and IGF-1R. Our data suggest that the TNF-α/NF-κB signaling pathway was upregulated when PDXs became resistant to the inhibition of the PI3K/AKT and IGF-1R/IR pathways. TNF-α signaling can exert both pro-tumorigenic and antitumorigenic effects depending on the context [42]. TNF-α can promote cancer cell survival, proliferation, and invasion by activating downstream signaling pathways, such as NF-kB and Mitogen-activated protein kinase (MAPK). In contrast, TNF-α can also induce cancer cell death through apoptosis under certain conditions [42]. The link between PI3K/AKT and TNF-α pathways lies in their crosstalk and reciprocal regulation. TNF-α can activate the PI3K/AKT pathway indirectly through the activation of receptor tyrosine kinases (RTKs) or directly through the activation of downstream signaling molecules. In addition, NF-kB can upregulate anti-apoptotic genes, such as Bcl-2 and Bcl-xL, protecting cancer cells from undergoing apoptosis induced by PI3K/AKT inhibition. Conversely, components of the PI3K/AKT pathway can modulate TNF-α signaling by regulating the expression of TNF receptors or downstream signaling molecules involved in TNF-α-induced responses [43,44]. In lung cancer, dysregulation of both pathways can lead to tumor progression, drug resistance, and poor clinical outcomes. Understanding the intricate interplay between the PI3K/AKT and TNF-α pathways is essential for developing targeted therapies and combination treatment strategies to effectively manage lung cancer and delay the development of resistance. A previous study showed that NF-κB activation by TNF requires AKT serine-threonine kinase [44]. Therefore, AKT plays a role in the signaling pathway necessary for inducing key immune and inflammatory responses [44]. Tumor necrosis factor α-induced protein 3 (TNFAIP3) is a protein triggered by TNF-mediated activation of NF-κB and plays a dual role in regulating this pathway. TNFAIP3 is linked to inflammation-driven carcinogenesis in various cancers [45]. Our molecular analyses revealed the modulation of hallmark pathways, particularly TNF-α signaling *via* NF-κB, PI3K/AKT, and inflammatory responses suppression.

While our preclinical data show robust therapeutic effects, it is important to recognize that cell lines and PDX models may not fully replicate the complexity of human tumor microenvironments. Further validation of these findings in clinical trials with larger cohorts of NSCLC patients is needed to confirm the efficacy and safety of this combination therapy. Identifying biomarkers to predict patient response will be critical for tailoring treatments to individual patients. Future studies should also explore potential combination therapies with other agents to enhance treatment response.

## Conclusion

In summary, our study provides robust preclinical evidence supporting the combination of a PI3K/AKT with an IGF-1R/IR inhibitor as a promising therapeutic strategy for NSCLC carrying PI3K/AKT pathway alteration. By targeting compensatory resistance mechanisms, this dual-pathway approach offers a potential therapeutic option for patients with advanced NSCLC, particularly LSCC. Importantly, this study is among the first to show synergistic activity specifically in NSCLC harboring activating alterations of the PI3K/AKT pathway. To translate these findings, prospective clinical trials are warranted to evaluate safety, efficacy, and biomarker-guided patient selection in larger population. Continued advances in targeted therapy, precision oncology, and resistance-mechanism research will be pivotal for improving patient outcomes in NSCLC and for further elucidating the molecular mechanisms underlying resistance.

## Data sharing statement

Data supporting the findings of this study are available within the article and its supplementary materials. Additional data are obtained from the corresponding author upon request.

## CRediT authorship contribution statement

**Mohamed Abd El-Salam:** Writing – review & editing, Conceptualization, Methodology, Investigation, Formal analysis, Data curation, Software, Validation, Visualization, Writing – original draft, Writing – review & editing, Project administration. **Wu Chen:** Conceptualization, Methodology, Investigation, Formal analysis, Data curation, Software, Validation, Visualization, Writing – review & editing, Project administration. **Yan Tang:** Methodology, Formal analysis. **Ting Rao:** Conceptualization, Methodology, Writing – review & editing. **Xuejia Kang:** Methodology, Formal analysis. **Lifang Sun:** Writing – review & editing. **Tiegang Han:** Methodology, Formal analysis. **Pengyu Chen:** Writing – review & editing. **Matthew Mossanen:** Conceptualization, Methodology, Writing – review & editing. **Fan Cheng:** Conceptualization, Methodology, Writing – review & editing. **Chun Yang:** Conceptualization, Methodology, Project administration, Writing – review & editing. **Chong-Xian Pan:** Conceptualization, Methodology, Supervision, Project administration, Writing – original draft, Writing – review & editing.

## Declaration of competing interest

The authors declare that they have no known competing financial interests or personal relationships that could have appeared to influence the work reported in this paper.

## Appendix Supplementary materials

Image, application 1

Image, application 2

Image, application 3
