# Supplementary material for: Synergistic effects through targeting the PI3K and IGFR pathways in treating lung cancer carrying activation alterations along the PI3K pathway
Source: Transl Oncol. 2026 Apr 3;67:102753. doi: 10.1016/j.tranon.2026.102753 (PMC13085011; doi:10.1016/j.tranon.2026.102753)
Supplement: Supplementary file 1 [file mmc1.pptx]

## Slide 1
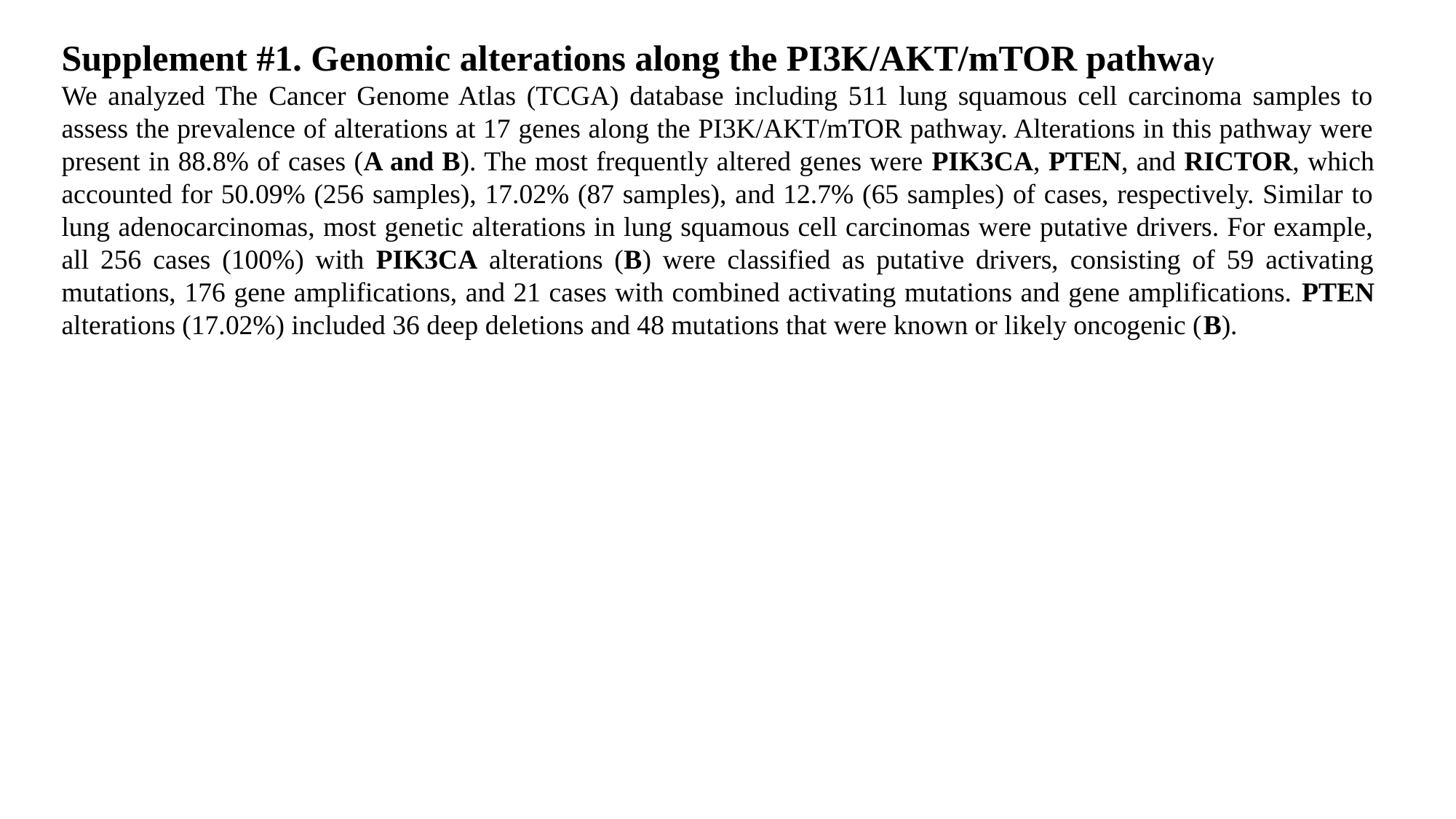

Supplement #1. Genomic alterations along the PI3K/AKT/mTOR pathway
We analyzed The Cancer Genome Atlas (TCGA) database including 511 lung squamous cell carcinoma samples to assess the prevalence of alterations at 17 genes along the PI3K/AKT/mTOR pathway. Alterations in this pathway were present in 88.8% of cases (A and B). The most frequently altered genes were PIK3CA, PTEN, and RICTOR, which accounted for 50.09% (256 samples), 17.02% (87 samples), and 12.7% (65 samples) of cases, respectively. Similar to lung adenocarcinomas, most genetic alterations in lung squamous cell carcinomas were putative drivers. For example, all 256 cases (100%) with PIK3CA alterations (B) were classified as putative drivers, consisting of 59 activating mutations, 176 gene amplifications, and 21 cases with combined activating mutations and gene amplifications. PTEN alterations (17.02%) included 36 deep deletions and 48 mutations that were known or likely oncogenic (B).

## Slide 2
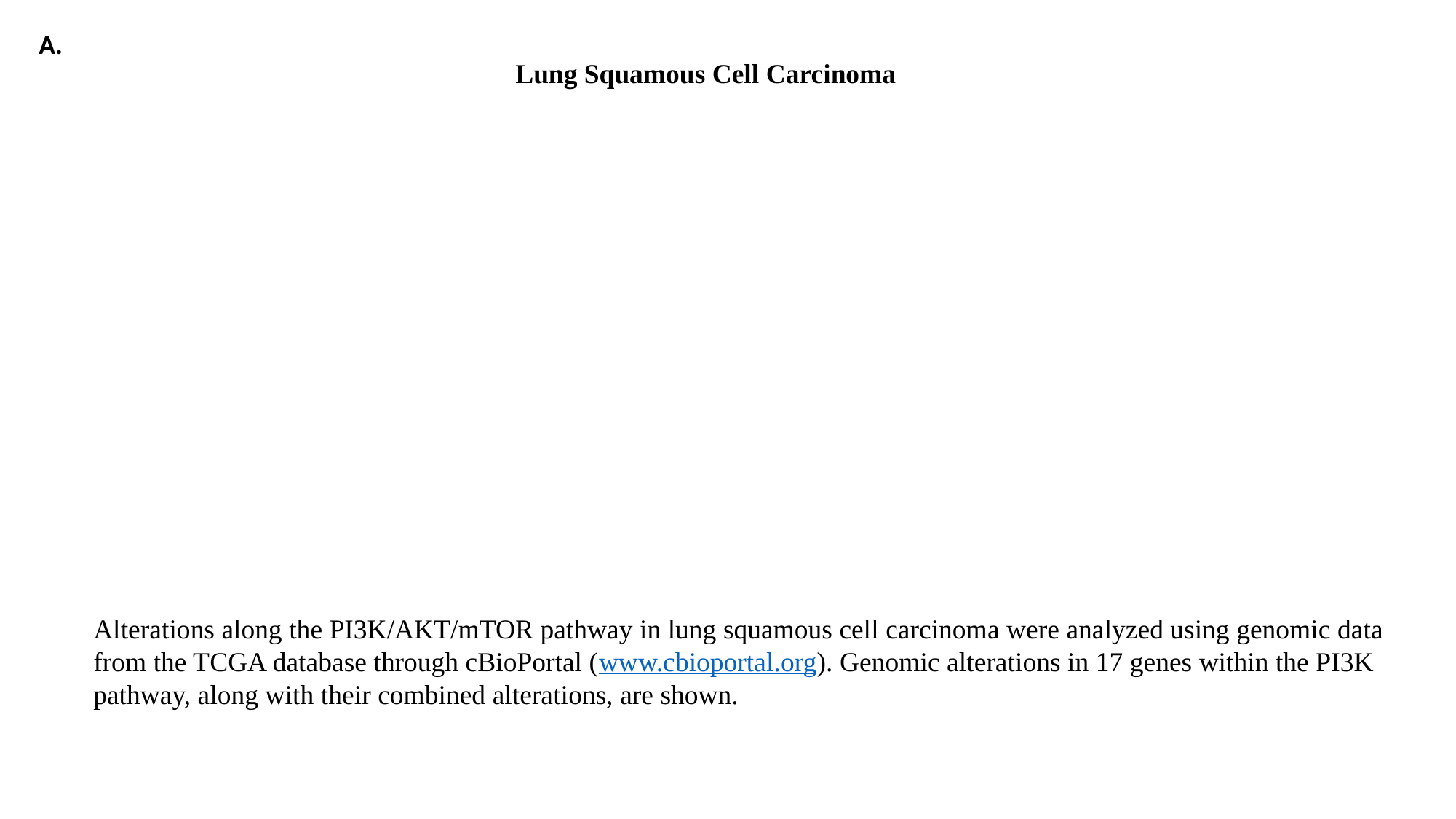

Lung Squamous Cell Carcinoma
A.
Alterations along the PI3K/AKT/mTOR pathway in lung squamous cell carcinoma were analyzed using genomic data from the TCGA database through cBioPortal (www.cbioportal.org). Genomic alterations in 17 genes within the PI3K pathway, along with their combined alterations, are shown.

## Slide 3
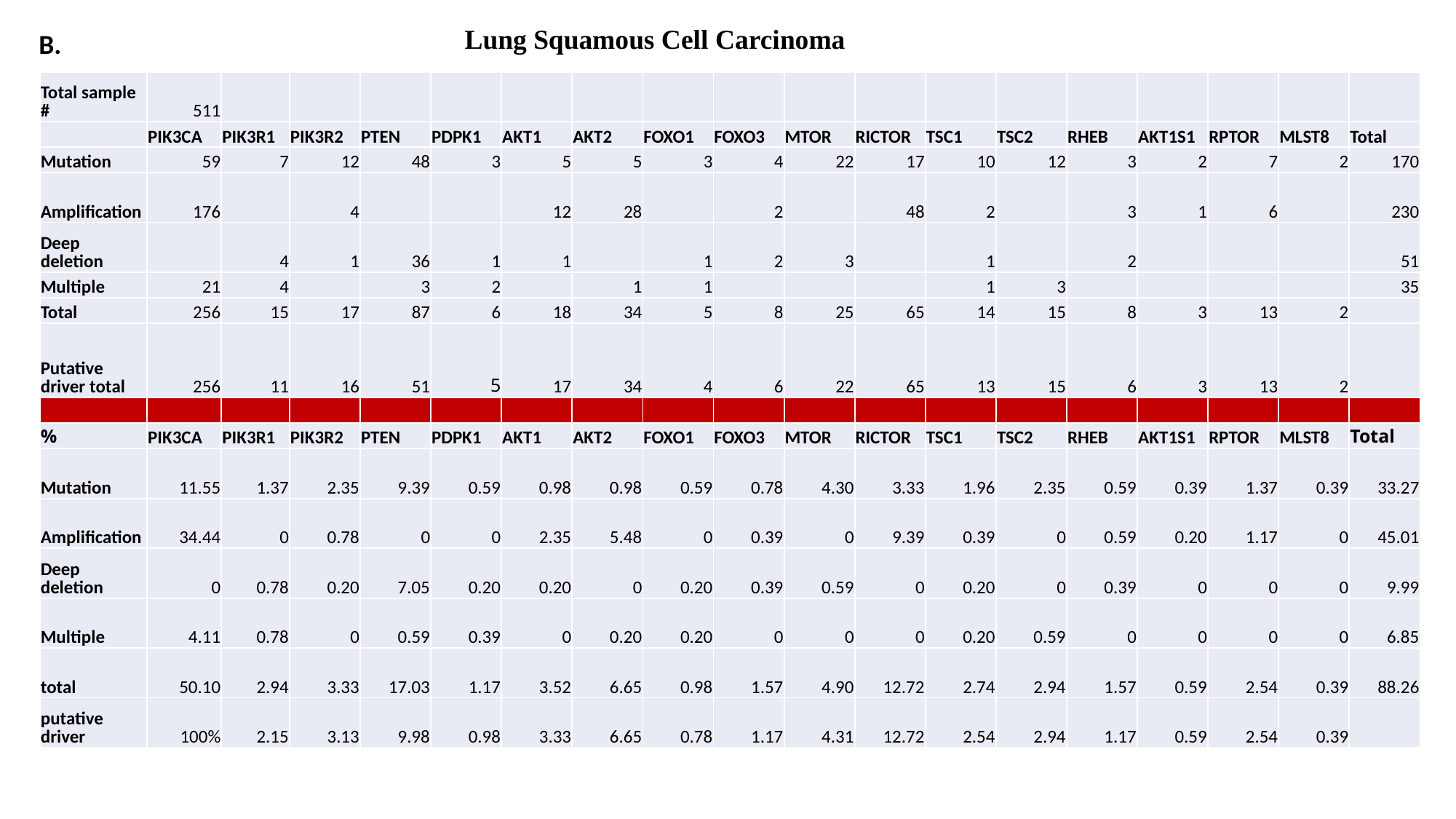

Lung Squamous Cell Carcinoma
B.
| Total sample # | 511 | | | | | | | | | | | | | | | | | |
| --- | --- | --- | --- | --- | --- | --- | --- | --- | --- | --- | --- | --- | --- | --- | --- | --- | --- | --- |
| | PIK3CA | PIK3R1 | PIK3R2 | PTEN | PDPK1 | AKT1 | AKT2 | FOXO1 | FOXO3 | MTOR | RICTOR | TSC1 | TSC2 | RHEB | AKT1S1 | RPTOR | MLST8 | Total |
| Mutation | 59 | 7 | 12 | 48 | 3 | 5 | 5 | 3 | 4 | 22 | 17 | 10 | 12 | 3 | 2 | 7 | 2 | 170 |
| Amplification | 176 | | 4 | | | 12 | 28 | | 2 | | 48 | 2 | | 3 | 1 | 6 | | 230 |
| Deep deletion | | 4 | 1 | 36 | 1 | 1 | | 1 | 2 | 3 | | 1 | | 2 | | | | 51 |
| Multiple | 21 | 4 | | 3 | 2 | | 1 | 1 | | | | 1 | 3 | | | | | 35 |
| Total | 256 | 15 | 17 | 87 | 6 | 18 | 34 | 5 | 8 | 25 | 65 | 14 | 15 | 8 | 3 | 13 | 2 | |
| Putative driver total | 256 | 11 | 16 | 51 | 5 | 17 | 34 | 4 | 6 | 22 | 65 | 13 | 15 | 6 | 3 | 13 | 2 | |
| | | | | | | | | | | | | | | | | | | |
| % | PIK3CA | PIK3R1 | PIK3R2 | PTEN | PDPK1 | AKT1 | AKT2 | FOXO1 | FOXO3 | MTOR | RICTOR | TSC1 | TSC2 | RHEB | AKT1S1 | RPTOR | MLST8 | Total |
| Mutation | 11.55 | 1.37 | 2.35 | 9.39 | 0.59 | 0.98 | 0.98 | 0.59 | 0.78 | 4.30 | 3.33 | 1.96 | 2.35 | 0.59 | 0.39 | 1.37 | 0.39 | 33.27 |
| Amplification | 34.44 | 0 | 0.78 | 0 | 0 | 2.35 | 5.48 | 0 | 0.39 | 0 | 9.39 | 0.39 | 0 | 0.59 | 0.20 | 1.17 | 0 | 45.01 |
| Deep deletion | 0 | 0.78 | 0.20 | 7.05 | 0.20 | 0.20 | 0 | 0.20 | 0.39 | 0.59 | 0 | 0.20 | 0 | 0.39 | 0 | 0 | 0 | 9.99 |
| Multiple | 4.11 | 0.78 | 0 | 0.59 | 0.39 | 0 | 0.20 | 0.20 | 0 | 0 | 0 | 0.20 | 0.59 | 0 | 0 | 0 | 0 | 6.85 |
| total | 50.10 | 2.94 | 3.33 | 17.03 | 1.17 | 3.52 | 6.65 | 0.98 | 1.57 | 4.90 | 12.72 | 2.74 | 2.94 | 1.57 | 0.59 | 2.54 | 0.39 | 88.26 |
| putative driver | 100% | 2.15 | 3.13 | 9.98 | 0.98 | 3.33 | 6.65 | 0.78 | 1.17 | 4.31 | 12.72 | 2.54 | 2.94 | 1.17 | 0.59 | 2.54 | 0.39 | |

## Slide 4
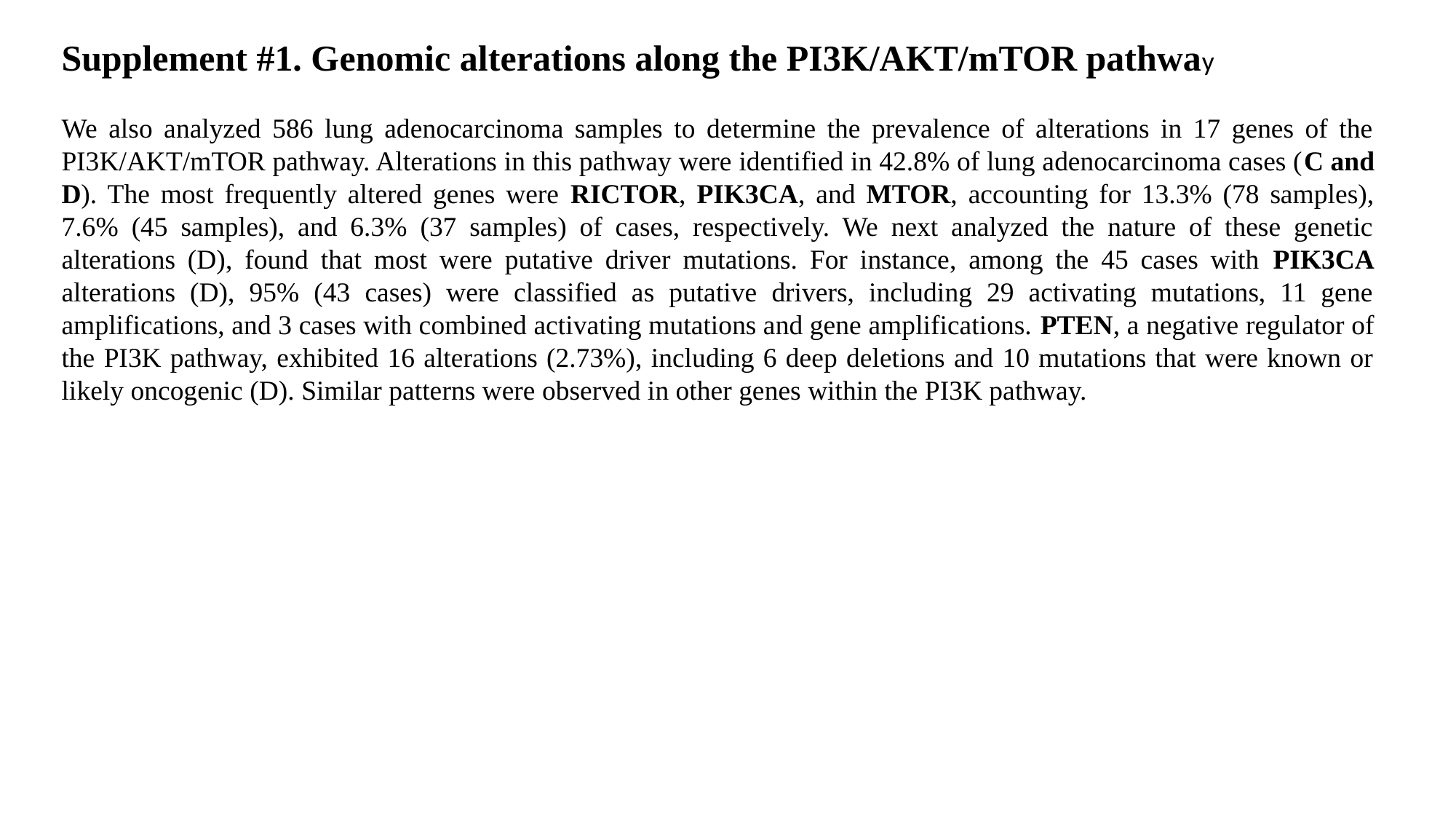

Supplement #1. Genomic alterations along the PI3K/AKT/mTOR pathway
We also analyzed 586 lung adenocarcinoma samples to determine the prevalence of alterations in 17 genes of the PI3K/AKT/mTOR pathway. Alterations in this pathway were identified in 42.8% of lung adenocarcinoma cases (C and D). The most frequently altered genes were RICTOR, PIK3CA, and MTOR, accounting for 13.3% (78 samples), 7.6% (45 samples), and 6.3% (37 samples) of cases, respectively. We next analyzed the nature of these genetic alterations (D), found that most were putative driver mutations. For instance, among the 45 cases with PIK3CA alterations (D), 95% (43 cases) were classified as putative drivers, including 29 activating mutations, 11 gene amplifications, and 3 cases with combined activating mutations and gene amplifications. PTEN, a negative regulator of the PI3K pathway, exhibited 16 alterations (2.73%), including 6 deep deletions and 10 mutations that were known or likely oncogenic (D). Similar patterns were observed in other genes within the PI3K pathway.

## Slide 5
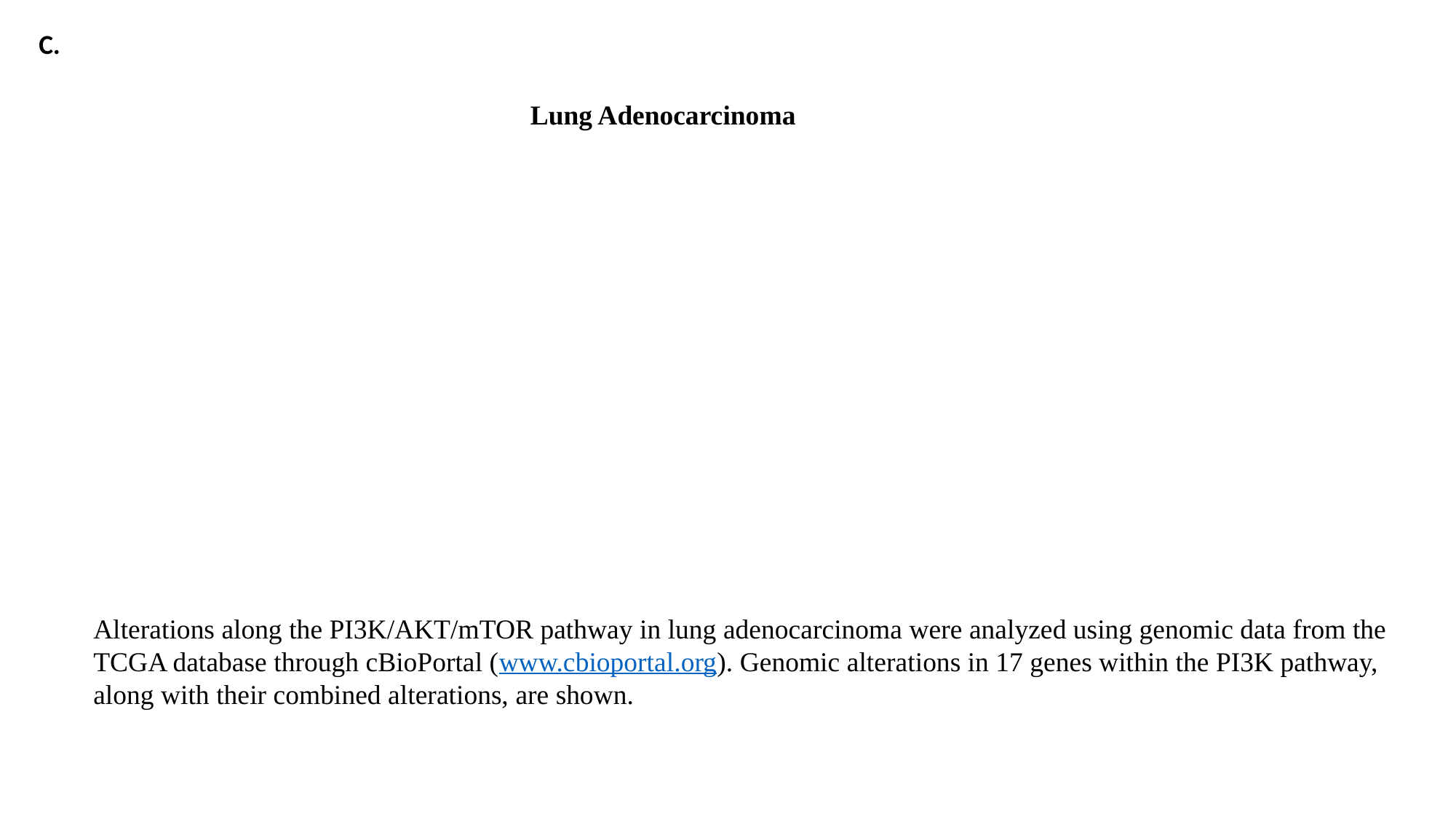

C.
Lung Adenocarcinoma
Alterations along the PI3K/AKT/mTOR pathway in lung adenocarcinoma were analyzed using genomic data from the TCGA database through cBioPortal (www.cbioportal.org). Genomic alterations in 17 genes within the PI3K pathway, along with their combined alterations, are shown.

## Slide 6
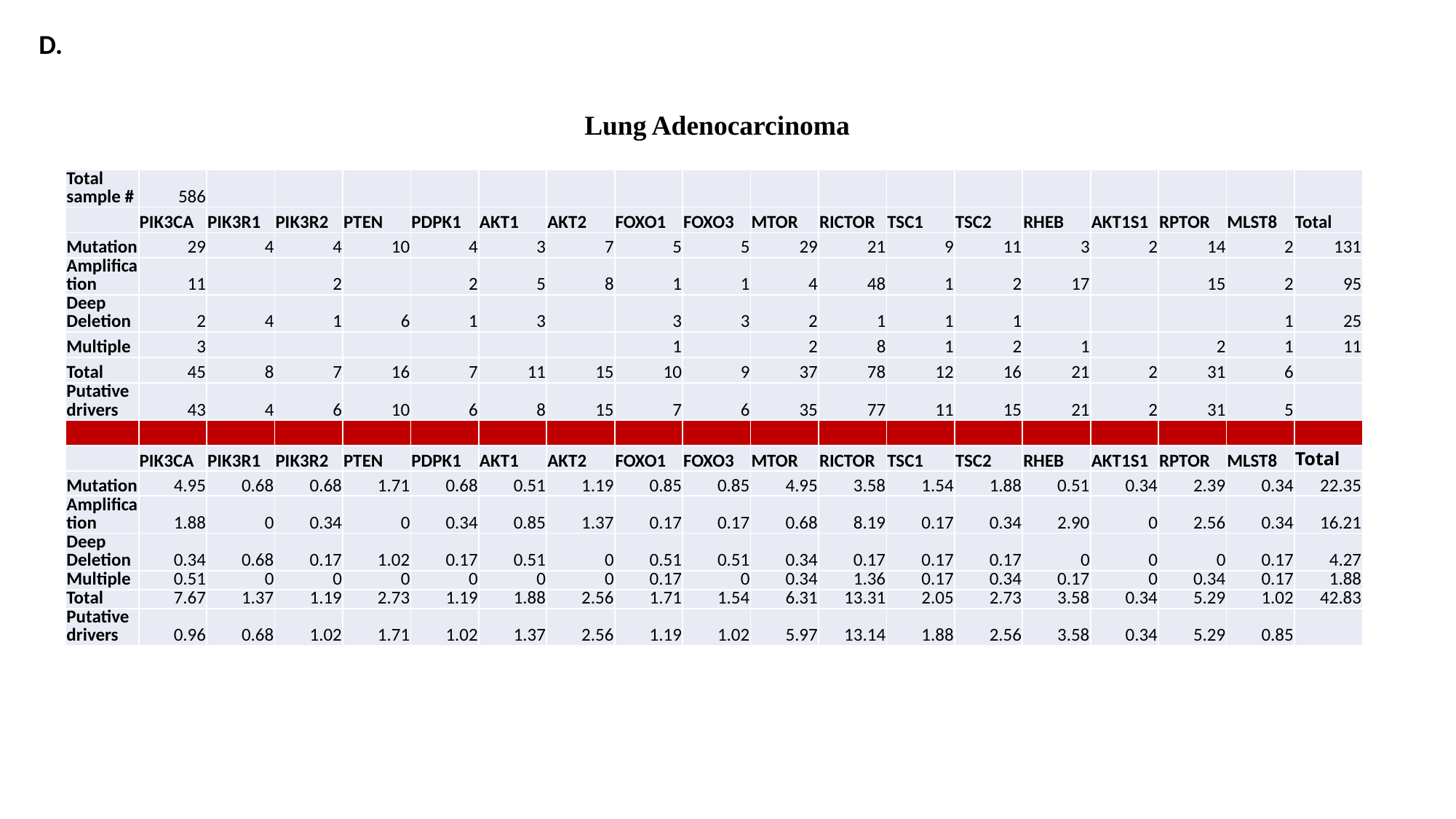

D.
Lung Adenocarcinoma
| Total sample # | 586 | | | | | | | | | | | | | | | | | |
| --- | --- | --- | --- | --- | --- | --- | --- | --- | --- | --- | --- | --- | --- | --- | --- | --- | --- | --- |
| | PIK3CA | PIK3R1 | PIK3R2 | PTEN | PDPK1 | AKT1 | AKT2 | FOXO1 | FOXO3 | MTOR | RICTOR | TSC1 | TSC2 | RHEB | AKT1S1 | RPTOR | MLST8 | Total |
| Mutation | 29 | 4 | 4 | 10 | 4 | 3 | 7 | 5 | 5 | 29 | 21 | 9 | 11 | 3 | 2 | 14 | 2 | 131 |
| Amplification | 11 | | 2 | | 2 | 5 | 8 | 1 | 1 | 4 | 48 | 1 | 2 | 17 | | 15 | 2 | 95 |
| Deep Deletion | 2 | 4 | 1 | 6 | 1 | 3 | | 3 | 3 | 2 | 1 | 1 | 1 | | | | 1 | 25 |
| Multiple | 3 | | | | | | | 1 | | 2 | 8 | 1 | 2 | 1 | | 2 | 1 | 11 |
| Total | 45 | 8 | 7 | 16 | 7 | 11 | 15 | 10 | 9 | 37 | 78 | 12 | 16 | 21 | 2 | 31 | 6 | |
| Putative drivers | 43 | 4 | 6 | 10 | 6 | 8 | 15 | 7 | 6 | 35 | 77 | 11 | 15 | 21 | 2 | 31 | 5 | |
| | | | | | | | | | | | | | | | | | | |
| | PIK3CA | PIK3R1 | PIK3R2 | PTEN | PDPK1 | AKT1 | AKT2 | FOXO1 | FOXO3 | MTOR | RICTOR | TSC1 | TSC2 | RHEB | AKT1S1 | RPTOR | MLST8 | Total |
| Mutation | 4.95 | 0.68 | 0.68 | 1.71 | 0.68 | 0.51 | 1.19 | 0.85 | 0.85 | 4.95 | 3.58 | 1.54 | 1.88 | 0.51 | 0.34 | 2.39 | 0.34 | 22.35 |
| Amplification | 1.88 | 0 | 0.34 | 0 | 0.34 | 0.85 | 1.37 | 0.17 | 0.17 | 0.68 | 8.19 | 0.17 | 0.34 | 2.90 | 0 | 2.56 | 0.34 | 16.21 |
| Deep Deletion | 0.34 | 0.68 | 0.17 | 1.02 | 0.17 | 0.51 | 0 | 0.51 | 0.51 | 0.34 | 0.17 | 0.17 | 0.17 | 0 | 0 | 0 | 0.17 | 4.27 |
| Multiple | 0.51 | 0 | 0 | 0 | 0 | 0 | 0 | 0.17 | 0 | 0.34 | 1.36 | 0.17 | 0.34 | 0.17 | 0 | 0.34 | 0.17 | 1.88 |
| Total | 7.67 | 1.37 | 1.19 | 2.73 | 1.19 | 1.88 | 2.56 | 1.71 | 1.54 | 6.31 | 13.31 | 2.05 | 2.73 | 3.58 | 0.34 | 5.29 | 1.02 | 42.83 |
| Putative drivers | 0.96 | 0.68 | 1.02 | 1.71 | 1.02 | 1.37 | 2.56 | 1.19 | 1.02 | 5.97 | 13.14 | 1.88 | 2.56 | 3.58 | 0.34 | 5.29 | 0.85 | |
