# Supplementary material for: Synergistic effects through targeting the PI3K and IGFR pathways in treating lung cancer carrying activation alterations along the PI3K pathway
Source: Transl Oncol. 2026 Apr 3;67:102753. doi: 10.1016/j.tranon.2026.102753 (PMC13085011; doi:10.1016/j.tranon.2026.102753)
Supplement: Supplementary file 2 [file mmc2.pptx]

## Slide 1
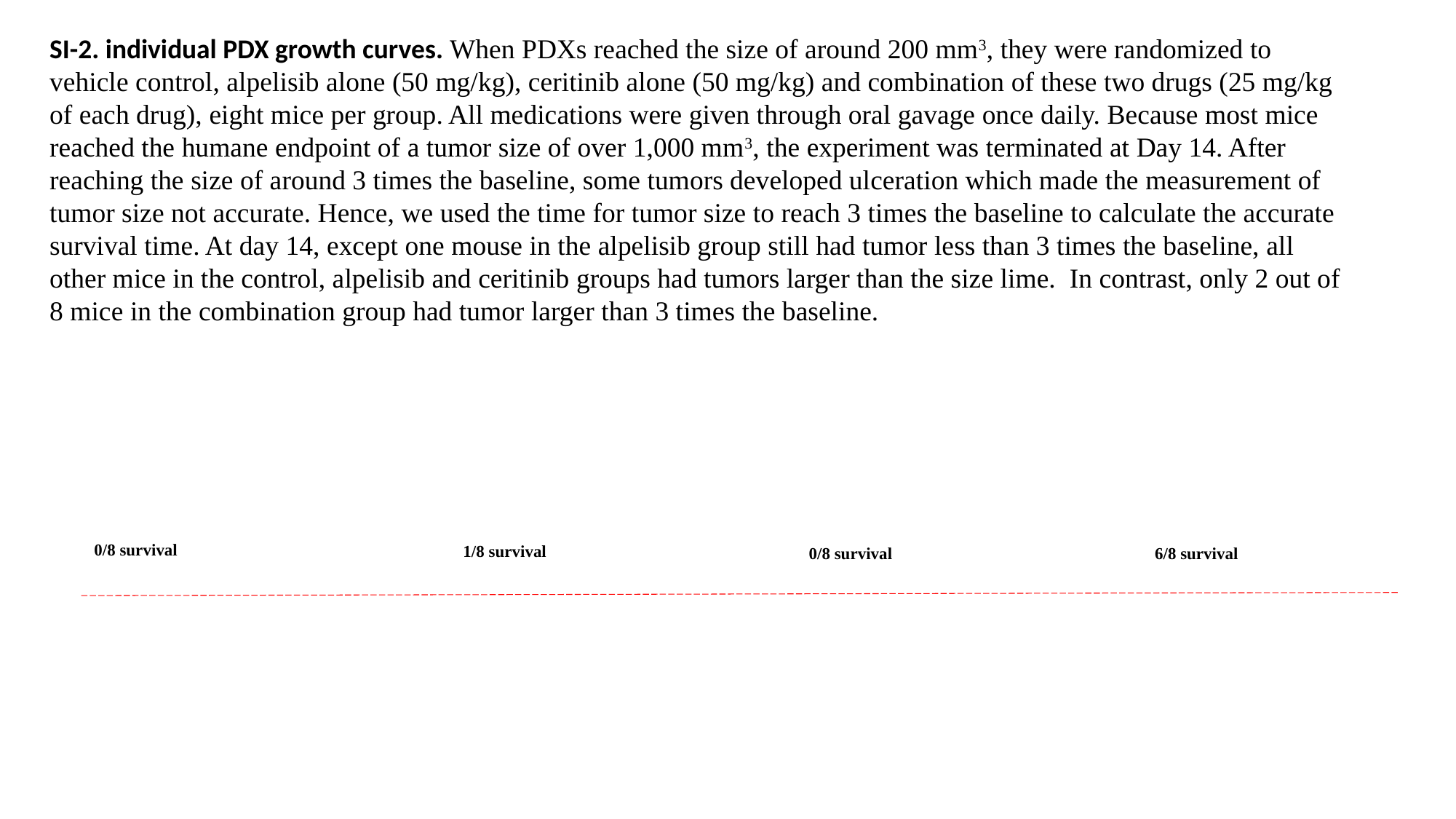

SI-2. individual PDX growth curves. When PDXs reached the size of around 200 mm3, they were randomized to vehicle control, alpelisib alone (50 mg/kg), ceritinib alone (50 mg/kg) and combination of these two drugs (25 mg/kg of each drug), eight mice per group. All medications were given through oral gavage once daily. Because most mice reached the humane endpoint of a tumor size of over 1,000 mm3, the experiment was terminated at Day 14. After reaching the size of around 3 times the baseline, some tumors developed ulceration which made the measurement of tumor size not accurate. Hence, we used the time for tumor size to reach 3 times the baseline to calculate the accurate survival time. At day 14, except one mouse in the alpelisib group still had tumor less than 3 times the baseline, all other mice in the control, alpelisib and ceritinib groups had tumors larger than the size lime. In contrast, only 2 out of 8 mice in the combination group had tumor larger than 3 times the baseline.
0/8 survival
1/8 survival
0/8 survival
6/8 survival
